# Health Disparities Research With the National Institute on Aging (NIA)

**DOI:** 10.1093/geroni/igab046.1400

**Published:** 2021-12-17

**Authors:** Cerise Elliott, Patricia Jones, Patricia Jones

## Abstract

The National Institute on Aging has taken special efforts to support research endeavors that explore ways to address health disparities. For example, the NIA Health Disparities Research Framework was developed in 2015 to provide a visualization of priority areas in Aging Research. The Framework can help researchers assess advances and potential opportunities for stimulating and supporting rigorous methods to address health disparities in Aging Research among the phases of research. The goal of this symposium is to highlight the different resources and research opportunities that NIA offers to support aging researchers, centers, and institutions for health disparities-related research or programs. Dr. Ron Kohanski will present a concept piece on how laboratory animals might be leveraged to mimic the impact of early life disparities on aging over the life-course, based on research in the hallmarks of aging support by NIA’s Extramural Division of Aging Biology. Dr. Damali Martin will identify the different resources focused on health disparities related research within the Division of Neuroscience. Dr. Lyndon Joseph will discuss the different health disparities research opportunities that are available from the Division of Geriatrics and Clinical Geriatrics. Dr. Frank Bandiera will highlight the different resources and research opportunities that are available to address health disparities within the Division of Behavior and Social Research. These presentations, taken together, will provide important information that bolsters knowledge of resources and research opportunities to address health disparities over the lifecourse and in late life.

